# Supplementary material for: Association of ABO blood groups with presentation and outcomes of confirmed SARS CoV-2 infection: A prospective study in the largest COVID-19 dedicated hospital in Bangladesh
Source: PLoS One. 2021 Apr 7;16(4):e0249252. doi: 10.1371/journal.pone.0249252 (PMC8026078; doi:10.1371/journal.pone.0249252)
Supplement: S1 File — (DOCX) [file pone.0249252.s001.docx]

**Research Protocol**

**Association of ABO blood groups with presentation and outcomes of confirmed SARS CoV-2 infection: A prospective study in the largest COVID-19 dedicated hospital in Bangladesh**

**Principal Investigator**

**Dr. Reaz Mahmud**

**FCPS (Medicine), MD (Neurology)**

**Assistant Professor**

**Department of Neurology**

**Dhaka Medical College, Dhaka**

**Part A**

**Project Title: Association of ABO blood groups with presentation and outcomes of confirmed SARS CoV-2 infection: A prospective study in the largest COVID-19 dedicated hospital in Bangladesh**

Principal Investigator:

| Name | Designations & place of posting |
| --- | --- |
| Dr. Reaz Mahmud | Assistant Professor, Department of Neurology, Dhaka Medical College |

2. **Co-Principal investigators:**

| Name | Designations & place of posting |
| --- | --- |
| Professor Mujibur Rahman | Professor and Head, Department of Medicine, Dhaka Medical College |

1. **Co-investigators:**

| Name | Designations& place of posting |
| --- | --- |
| Dr. S. K Jakaria Been Sayeed | Indoor Medical Officer, Department of Medicine, Dhaka Medical College. |
| Dr.MD. Shahidul Islam | Junior Consultant, Medecine, Sarkari Karmachari Hospital, Dhaka. |
| Dr. Mohammad Aftab Rassel | Medical officer, OSD, MD Thesis part student, Department of Neurology, Dhaka Medical College |
| Dr. Farhana Binte Monayem | Medical Officer, Sarkari Karmachari Hospital,Dhaka. |
| DR. Mohammed Monirul Islam | Assistant Surgeon, MoHFW, Dhaka, Bangladesh |
| Dr. Mohammad Abdullah Yusuf | Assistant Professor, Department of Microbiology, National Institute of Neurosciences and Hospital, Dhaka. |
| Dr. KM Nazmul Islam | Assistant professor, Department of Neurology, Shaheed Suhrawardy Medical College, Dhaka, |
| Dr. Mohammad Zaid Hossain | Associate professor, Department of Medicine, Dhaka Medical College Hospital, Dhaka. |
| Dr. A.K.M. Humayon Kabir | Associate professor, Department of Medicine, Dhaka Medical College Hospital, Dhaka. |
| Dr. Ahmed Hossain Chowdhury | Associate Professor, Dept. of Neurology, Dhaka Medical College Hospital, Dhaka |
| Dr. Sabrina Rahman | Post graduate Trainee, Medicine department, Dhaka Medical College Hospital |
| Dr. Kazi Gias Uddin Ahmed | Associate Professor and Head, Department of Neurology, Dhaka Medical College |
| Dr. Imran Mahmud | Registrar, Department of Medicine, Dhaka Medical College |

1. Place of the study/Institution(s):

COVID-19 Unit, Dhaka Medical College Hospital.

1. Sponsoring/Collaborating Agencies:

None

1. Duration:

04 months

1. Date of Commencement:

June 2020

1. Date of Completion:

September 2020

1. Total Cost: Not estimated

10. Other Support for Proposed Research:

(1) Is this research project being No

supported by any other source?

(2) Has an application for funding of No

this project been submitted to any

other organization(s)?

11. Date of Submission : 04-10-2020

12. Signature of Principal Investigator(s) :

| Name | Designations & place of posting | Signature |
| --- | --- | --- |
| Dr. Reaz Mahmud | Assistant Professor, Department of Neurology, Dhaka Medical college |  |

**PART – B**

**PRINCIPAL INVESTIGATOR(S) INFORMATION SHEET**

1. (i) Name :**Dr. Reaz Mahmud**

(ii) Designation: Assistant Professor, Neurology

(iii) Official Address with telephone: Dhaka Medical College Hospital, Dhaka.

Phone: 01912270803

(iv) Present Residential Address with telephone: Road # 07, House # 13

Abdullah bagh, Uttar Badda, Badda Dhaka.

1. **Academic Background:**

| Degree | University | Field | Year |
| --- | --- | --- | --- |
| MBBS | Dhaka University |  | May,2003 |
| FCPS | BCPS | Medicine | January,2013 |
| MD | Dhaka University | Neurology | June,2015 |

1. **Field of Specialty:**

Medicine, Neurology, Research Methodology and Data Analysis

1. **Membership:** Bangladesh Society of Medicine, Society of Neurologists Bangladesh, International Headache society, Asia Pacific Headache Society.
2. **(a) Research Experience :**

| 1. **Association between Hypertensive retinopathy and Lacunar stroke.** | Done for FCPS Dissertation |
| --- | --- |
| 2. **Risk Factors and Morphological Differences of Ruptured Saccular Aneurysm in different sites of Anterior Circulation in Patients presenting with Subarachnoid Haemorrhage** | Done for MD thesis |
| 3**. Recent sensitivity Pattern of Salmonella Typhi in a private hospital.** | Done for Research |
| 4. A Randomized, Double-Blind Placebo Controlled Clinical Trial of Ivermectin plus Doxycycline for the Treatment of Confirmed Covid -19 Infection | Completed Research |

**(b) Other Experience:**

**Teaching:**

1. Assistant Registrar cardiology NICVD, 03-08-09 to 21-06-10
2. Assistant Registrar Medicine, Faridpur Medical college Hospital, Faridpur 23-06-10 to 31-06-12

**Administration:**

Work as a Departmental Head, Critical Care Medicine, Sarkari Karmachari Hospital from 01-11-2016 to 04-12-2019

5. **Number of Scientific Publications:**

| Sl No | National/  International | Original/  review | Authorship | Reference |
| --- | --- | --- | --- | --- |
| 1 | National | Original | Co-author | Ali M Y, Mahmud R. A case report on Lepra Reaction Type II. Faridpur Medical college journal 2012; 2 : 93-97 |
| 2 | National | Original | Author | Mahmud R, Ali MY, Islam MS, Shanewaz S, Rabbani G, Manayem FB. Association Between Hypertensive Retinopathy and Lacunar Infarct- A Study in Faridpur Medical College Hospital. DCIMC J 2016; 3(2):27-33 |
| 3 | National | Original | Co-author | Saha R, Mahmud R, Hossain MZ,Sarker PK. Families with Neurocutaneous Syndrome:Report of two cases.Dhaka Med Coll J 2013; 22(1): 102-107. |
| 4 | National | Original | Author | Mahmud R, Habib M, Uddin S,Risk Factors and Morphological Differences of Ruptured Saccular Aneurysm in Different Sites of Anterior Circulation in Patients Presenting with Subarachnoid Haemorrhage. Journal of National Institute of Neurosciences Bangladesh. January 2017; 3(1):21-28. |
| 5 | National | Original | Author | Islam K, Mahmud R. Recent sensitivity Pattern of Salmonell Typhi in a private hospital.J Medicine 2018; 19: 15-17 |
| 6 | National | Original | Author | Mahmud R, Habib M. Huntington's Disease with Retinitis Pigmentosa- a Case Report. Faridpur Med. Coll. J 2017; 12(1):50-52. |
| 7 | National | Original | Co-Author | Ghose, S., Ahmed, K. G., Chowdhury, A., Hasan, A., Saha, K., Mahmud, R., Joy, N., Biswas, R., Sarkar, M. S., Rahman, M. M., Sina, H., Arifuzzaman, M., Alam, I., Hossain, M. M., Karim, A., & Habib, M. (2018). Assessment of Initial Stroke Severity by National Institute Health Stroke Scale (NIHSS) Score at Admission. *Journal of Dhaka Medical College*, *26*(2), 90-93. https://doi.org/10.3329/jdmc.v26i2.38765 |

Signature of Principal Investigator


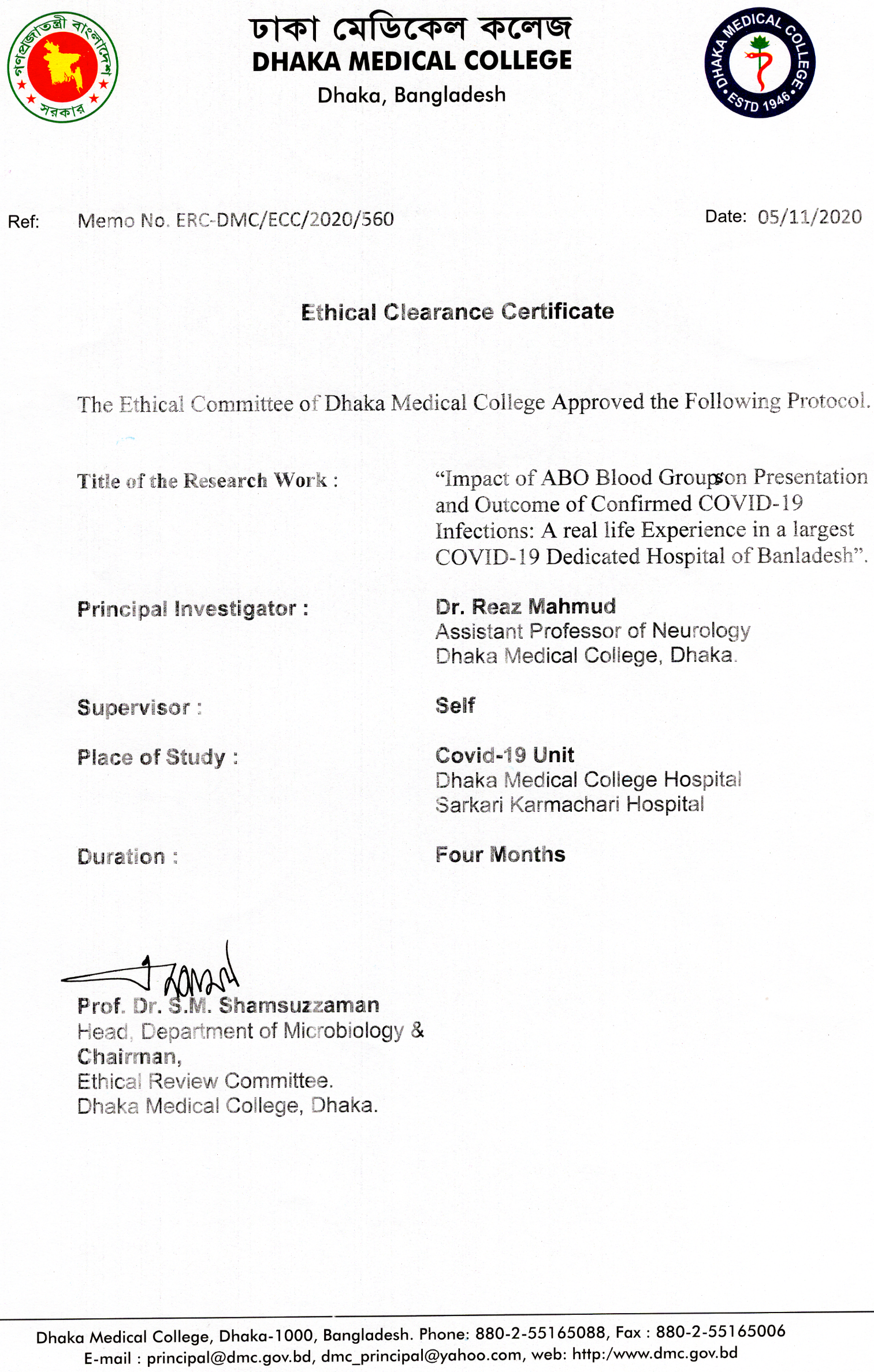


**PART - C**

**Project Title: Association of ABO blood groups with presentation and outcomes of confirmed SARS CoV-2 infection: A prospective study in the largest COVID-19 dedicated hospital in Bangladesh**

**Summary**:

Since December 2019 COVID-19 is dominating the life of the people of this universe. Its behavior also varied among the nations of the universe. The risk factor of adverse outcome of COVID-19 was determined. No biologic markers for the susceptibility of the infection is yet to be found. The relation of blood group and infection was found in several disease like cholera, severe malaria, severity of dengue, H-pylori infection have been elucidated. Genome wide Association Study of Severe Covid-19 with Respiratory Failure, GWAS Group detected cross-replicating associations with rs11385942 at locus 3p21.31 and with rs657152 at locus 9q34.2. The association signal at locus 9q34.2 coincided with the ABO blood group locus. Several studies also found some contradicting results in this regard.

So to observe the impact of ABO blood groups on the presentation and outcome of Confirmed COVID -19 infection, this study was conducted in the largest COVID19 dedicated hospital in Bangladesh. It would be a prospective cohort study. A cohort of the patients with RT-PCR positive COVID-19 patients since June 2020 till September 2020 will be included in this cohort. Their blood group will be determined. The clinical features, severity, time required to recover, conversion of severity, Persistence of RT-PCR positivity 14 days after initial positivity will be determined. They will be followed up for at least 30 days or hospital discharge which one is longer. Comparison will be made in different blood groups.

The primary outcome would be the duration required to have clinical improvement.

Data will be analyzed using SPSS 20. To compare the groups one way ANOVA was done for continuous variable, Chi square for categorical variables. For outcome assessment relative risk (RR) with 95% CI was measured for qualitative variable categorizing blood group A and non A. survival analysis by Kaplan-Meier curve was done for the duration of recovery. Result of the study and statistical analysis will be presented by tables, figures, graphs, diagrams, charts and photographs. All these will have own legends (i.e. title) and will be serially numbered. Discussion will be done on the basis of result obtained from the study and comparing with similar studies done at home and abroad. Summarization will be drawn after discussion. Conclusion will be drawn depending upon the results and discussion.

**PART - D**

**Introduction:**

WHO (World Health Organization) first notify about the emergence of COVID-19 infection in Wuhan city, China in Late December^1^. Soon the novel virus became pandemic, declared on 12 March 2020^2^. On 19 September 2020, there have been 30,369,778 confirmed cases of COVID-19, including 948795 deaths reported to WHO^3^. The presentation of COVID-19 varied widely. The most common symptoms of COVID-19 involved fatigue, fever, dry cough, respiratory distress, anosmia and so on. A large proportion of the patient remains Asymptomatic as well^4^. Its behavior also varied among the nations of the universe. Genome wide Association Study on Severe Covid-19 with Respiratory Failure, GWAS Group detected cross-replicating associations with rs11385942 at locus 3p21.31 and with rs657152 at locus 9q34.2. The association signal at locus 9q34.2 coincided with the ABO blood group locus. They found a higher risk among persons with blood group A than with other blood groups^5^.Some other viruses like HBV^6^, SARS-COV^7^, and MERS-COV^8^ have susceptibility relation with ABO blood group. The mechanisms behind the impact of blood group are still unclear. Histo-blood group antigens are also expressed on endothelial cells (EC) and platelets^9^. SARS-CoV enter into human body through the replication in epithelial cells of the respiratory and digestive tracts. They have the ability to synthesize ABH carbohydrate epitopes. It has been hypothesized that the S protein of virions produced by either A or B individuals could be decorated with A or B carbohydrate epitopes, respectively. Guillon et al^10^. Reported that interaction between S protein and ACE-2 is specifically inhibited by human natural anti-A antibodies. SARS-COV and SARS-COV-2 have similar nucleic acid sequence and similar receptor combination with angiotensin-converting enzyme 2 (ACE2)^11^. It was also found that non-A type is a risk factor for venous thromboembolism, which is one of the pathogenesis for COVID-19 death^12^. But different studies showed contradicting results in relation to influence of blood group on susceptibility and outcome of COVID-19 infection^13, 14, 15, 16^. So to observe the impact of ABO blood groups on the presentation and outcome of Confirmed COVID -19 infection, this study was conducted in the largest COVID19 dedicated hospital in Bangladesh.

**2. OBJECTIVES:**

**General objectives:**

To observe the association of different blood groups on presentation and outcome of confirmed COVID-19 infection.

**Specific objectives:**

1. To observe the blood groups of the patients with COVID-19 infection.
2. To observe presentation and different outcome of the patients with confirmed COVID-19 infection.
3. To observe the differences in different blood groups.

**3. RATIONALE:**

Covid-19 is an emergent pandemic, threatens the life of millions of the people throughout the globe. The severity, outcome and susceptibility varies among the nations, race and individuals. Biologic markers of COVID-19 infection is still uncertain. Several studies revealed blood group A is most susceptible. The studies were performed with the affected patients only. This study Aim to observe the impact of different blood groups on presentation and outcome of confirmed COVID-19 infection. It would help us to detect a biologic marker for the susceptibility and outcome of COVID-19 infection.

1. **METHODOLOGY:**

Study type: Prospective cohort

Sample size:

Proportions to cure from COVID-19 by day-12 in group-1(blood group A) and group-2 (blood group Assuming expected B, O, AB) are 0.70 and 0.90 respectively. Thus we need a total of 378 samples at 1:2 ratio, and, that would provide a power of at least 90% at two tailed test where p value less than 0.05 to detect significant difference between the groups. Considering 10% dropout, we need 416 samples in total.

$$n=\frac{r+1}{r}\frac{p^{*}\left( 1-p^{*} \right){(Z_{\beta}+Z_{\frac{\alpha}{2}})}^{2}}{{{(p}_{1}- p_{2})}^{2}}$$

r = ratio of group-1 and group - 2

p*= Average of proportion

Z_α/2_ = Level of significance

Z_β_ = Power of the test

p_1_ - p_2_= Effect size

p_1_ = Proportion in group - 1

p_2_ = Proportion in group - 2

**Sampling: Consecutive sampling.**

Estimated study date: June, 2020

Estimated primary completion date, August 2020

Estimated study completion date: September, 2020

**Inclusion Criteria:**

- At least 18 years of age
- COVID-19 infection, confirmed by polymerase chain reaction (PCR) test
- Mild to moderate COVID-19 infection

**Exclusion Criteria:**

- Not willing to participate.
- Discontinue duties for any reason during the specified period
- Presence of haemoglobinopathies or other blood disorders.

**Research instruments:**

1. Informed consent form.

2. Case record form

**Primary Outcome Measure:**

- 1. Duration required to have clinical improvement

[Time Frame: 1 months]

**Secondary Outcome Measure:**

1. Conversion to the next level of severity

Time Frame: 1 month]

1. Persistent positivity 14 days after initial positivity

[Time Frame: 14 days]

1. Severity of the disease

[Time Frame: 1 month]

1. Development of post COVID syndrome

[Time Frame: 1 month]

**Data collection technique:**

Data will be collected by assigned trained data collectors (Physician).

Patient will be enrolled according to defined inclusion and exclusion criteria in the current research. Informed written consent will be obtained from the participants. Each participants participating in the trial will be uniquely identified, and information such as his name, address is recorded in the trial 'subject number list'.

**Data analysis**:

To compare the groups one way ANOVA was done for continuous variable, Chi square for categorical variables. For outcome assessment relative risk (RR) with 95% CI was measured for qualitative variable categorizing blood group-A and non-A (Group-B, AB, O). Survival analysis by Kaplan-Meier curve was done for the duration of recovery.

**Observation and Results:**

Result of the study and statistical analysis will be presented by tables, figures, graphs, diagrams, charts and photographs. All these would have own legends (i.e. title) and will be serially numbered.

**Discussion:**

Discussion will be done on the basis of result obtained from the study and comparing with similar studies done at home and abroad.

**Summary:**

Summarization will be drawn after discussion.

**Conclusion:**

Conclusion would be drawn depending upon the results and discussion

**Conflict of interest:** None

**Operational definition:**

**(According to WHO and National guideline)**

**Confirmed Covid-19:**

Cases with positive RT-PCR for Covid 19 irrespective of symptoms**.**

**Uncomplicated (mild) Illness**

These patients usually present with symptoms of an upper respiratory tract viral infection, including mild fever, cough (dry), sore throat, nasal congestion, malaise, headache, muscle pain, or malaise. Signs and symptoms of a more serious disease, such as dyspnea, are not present.

**Moderate Pneumonia**

Respiratory symptoms such as cough and shortness of breath (or tachypnea in children) are present without signs of severe pneumonia.

**Severe Pneumonia**

Fever is associated with severe dyspnea, respiratory distress, tachypnea (> 30 breaths/min), and hypoxia (SpO2 < 90% on room air). However, the fever symptom must be interpreted carefully as even in severe forms of the disease, it can be moderate or even absent. Cyanosis can occur in children. In this definition, the diagnosis is clinical, and radiologic imaging is used for excluding complications.

**Acute Respiratory Distress Syndrome (ARDS)**

The diagnosis requires clinical and ventilatory criteria. This syndrome is suggestive of a serious new-onset respiratory failure or for worsening of an already identified respiratory picture. Different forms of ARDS are distinguished based on the degree of hypoxia. The reference parameter is the PaO2/FiO2:

- Mild ARDS: 200 mmHg < PaO2/FiO2 ≤ 300 mmHg. In not-ventilated patients or in those managed through non-invasive ventilation (NIV) by using positive end-expiratorypressure (PEEP) or a continuous positive airway pressure (CPAP) ≥ 5 cmH2O.
- Moderate ARDS: 100 mmHg < PaO2/FiO2 ≤ 200 mmHg.
- Severe ARDS: PaO2/FiO2 ≤ 100 mmHg.

When PaO2 is not available, a ratio SpO2/FiO2 ≤ 315 is suggestive of ARDS.

Chest imaging utilized includes chest radiograph, CT scan, or lung ultrasound demonstrating bilateral opacities (lung infiltrates > 50%), not fully explained by effusions, lobar, or lung collapse.

**Clinical improvement Criteria**

1. Body temperature remains normal for at least 3 days (ear temperature is lower than 37.5 °C).

2. Respiratory symptoms are significantly improved.

3. Lung imaging shows obvious improvement in lesions.

4. There is no co-morbidities or complications which require hospitalization.

5. SpO2, >93% without assisted oxygen inhalation.

**5. UTILIZATION OF RESULTS:**

This study will observe the benefit of combined Doxycycline and Ivermectin on confirmed covid 19 patient. The study result will be compared with other trials in home and abroad.

**8. FLOW CHART of Study workup:**

| Sl No | **Activities** |  |  |  |  |  |  |
| --- | --- | --- | --- | --- | --- | --- | --- |
|  |  | **1^st^** | **2^nd^** | **3^rd^** | **4^th^** | **5^th^, to 15^th^** | **16^th^** |
| 1 | **Recruitment and training of the field staff** |  |  |  |  |  |  |
| 2 | **Pretesting and finalization of the questionnaire** |  |  |  |  |  |  |
| 3 | **Consultative meeting** |  |  |  |  |  |  |
| 4 | **Data collection** |  |  |  |  |  |  |
| 5 | **Data entry and editing** |  |  |  |  |  |  |
| 6 | **Data analysis and draft report writing** |  |  |  |  |  |  |
| 7 | **Dissemination of Results** |  |  |  |  |  |  |

1. **ETHICAL IMPLICATIONS**

The following points will be considered during the study:

1. Patients (subjects) and key relatives were clearly informed about the scope and limitation of the study.

2. Written consent will be obtained from the patients (subjects) or from parents if patient (subject) is unable to give reliable information.

3. Confidentiality of the patients (subjects) about personal information was strictly maintained.

4. The study will not be causing any environmental hazard.

**REFERENCES:**

1. World Health Organization. GCM teleconference – Note for the Records. 10 January 2020. Subject: Pneumonia in Wuhan, China. Available from: https: // www. WHO. int/ blueprint / 10-01-2020-nfr-gcm.pdf?ua=.
2. WHO Director-General's opening remarks at the media briefing on COVID-19 - 11 March 2020, available from:<https://www.who.int/dg/speeches/detail/who-director-general-s-opening-remarks-at-the-media-briefing-on-covid-19---11-march-2020>.
3. WHO Corona virus (Covid-19) Dashboard available from https: //extranet. who.int/ public emergency.
4. Rodriguez-Morales AJ, Cardona-Ospina JA, Gutie´rrez-Ocampo E, Villamizar-Pen˜a R, Holguin-Rivera Y, Escalera-Antezana JP, et al. Clinical, laboratory and imaging features of COVID-19: a systematic review and meta-analysis. Travel Med Infect Dis. 2020; 34:101623.
5. Ellinghaus D, Degenhardt F, Bujanda L, Buti M, Albillos A, Invernizzi P et al , Severe Covid-19 GWAS Group. Genomewide Association Study of Severe Covid-19 with Respiratory Failure. N Engl J Med. 2020 Jun 17:NEJMoa2020283. doi: 10.1056/NEJMoa2020283. Epub ahead of print. PMID: 32558485; PMCID: PMC7315890.
6. Jing W, Zhao S, Liu J*, Liu M .*ABO blood groups and hepatitis B virus infection: a systematic review and meta-analysis. *BMJ Open*2020; 10:e034114. doi: 10.1136/bmjopen-2019-034114
7. Patrice Guillon, Monique Clément, Véronique Sébille, Jean-Gérard Rivain, Chih-Fong Chou, Nathalie Ruvoën-Clouet, Jacques Le Pendu, Inhibition of the interaction between the SARS-CoV Spike protein and its cellular receptor by anti-histo-blood group antibodies, Glycobiology, Volume 18, Issue 12, December 2008, Pages 10851093, <https://doi.org/10.1093/glycob/cwn093>
8. Varughese, S., Read, J.G., Al-Khal, A., et al., 2015. Effectiveness of the Middle East respiratory syndrome-coronavirus protocol in enhancing the function of an Emergency Department in Qatar. Eur. J. Emerg. Med. 22 (5), 316–320.
9. Marionneau S, Cailleau-Thomas A, Rocher J, Le Moullac-Vaidye B, Ruvoen- ¨ clouet N, Clement M, Le Pendu J. ABH and Lewis histo-blood group ´ antigens, a model for the meaning of oligosaccharide diversity in the face of a changing world. Biochimie 2001; 83:565–573.
10. Guillon P, Clement M, Sebille V, Rivain JG, Chou CF, Ruvoen-Clouet N, et al. Inhibition of the interaction between the SARS-CoV spike protein and its cellular receptor by anti-histo-blood group antibodies. Glycobiology. 2008;18:1085–93
11. [Wan](https://scholar.google.com/citations?user=xsf8PVsAAAAJ&hl=en&oi=sra) Y,  [Shang](https://scholar.google.com/citations?user=0VnIEcUAAAAJ&hl=en&oi=sra) J.  [Graham](https://scholar.google.com/citations?user=QvEQLbAAAAAJ&hl=en&oi=sra) R, RS Baric RS, Li F. Receptor recognition by the novel coronavirus from Wuhan: an analysis based on decade-long structural studies of SARS coronavirus. J. Virol.2020. 94 (7).
12. Non-O Blood Type Is the Commonest Genetic Risk Factor for VTE: Results from a Meta-Analysis of the Literature Dentali Francesco, Sironi Anna, Ageno Walter, Turato Sara, Bonfanti Carlo, Frattini Francesco, Crestani Silvia, Franchini Massimo Seminars in Thrombosis and Hemostasis (2012-June-27) <https://doi.org/f33vdg> DOI: 10.1055/s-0032-1315758
13. Latz CA, DeCarlo C, Boitano L, Png CYM, Patell R, Conrad MF, Eagleton M, Dua A. Blood type and outcomes in patients with COVID-19. Ann Hematol. 2020 Sep; 99(9):2113-2118. doi: 10.1007/s00277-020-04169-1. Epub 2020 Jul 12. PMID: 32656591; PMCID: PMC7354354.
14. Zhao, J., Yang, Y., Huang, H.-P., et al., 2020. Relationship between the ABO Blood Group and the COVID-19 susceptibility. medRxiv.
15. Zietz, M., Tatonetti, N.P., 2020. Testing the association between blood type and COVID19 infection, intubation, and death. medRxiv
16. Zeng, X., Fan, H., Lu, D., et al., 2020. Association between ABO blood groups and clinical outcome of coronavirus disease 2019: evidence from two cohorts. medRxiv

**Abstract summary for Ethical Committee**

This hospital based Experimental study will be carried out in COVID-19 Unit, Dhaka Medical College Hospital for the duration of 4 months. Consecutive confirmed COVID-19 patients with mild to moderate severity whom will be enrolled from June, 2000 to august, 2000. Total 400 cases will be enrolled for the research. They will be followed up for at least 30 days or hospital discharge which one is longer.

1. By the following under mentioned steps confidentially will be maintained:

- Research data will be coded
- Data will be stored in a locked cabinets
- Only research personnel will be allowed to access data
- There is no physical, psychological, social and legal risk during physical examination. Proper consent will be taken.
- For safeguarding confidentially and protecting anonymity each of the patient will be given and ID no.
- A signed informed consent will be taken from the patient/patients guardians convincing that privacy of the patient will be maintained and he/she will not be compensated for loss of work time if they want.
- A data should (enclosed) be prepared for which a short interview of 15-30 minutes will be required
- Use of hospital records (indoor) will be needed to fill up the patients’ data sheet.

2. Consent form will be a written statement

1. A brief interview regarding study variables will be collected from the participants.

**Circle the appropriate answer to each of the following**

**(If not Applicable write NA).**

| **1. Source of population:** |  |  | **4. Are subjects clearly informed about** |  |  |
| --- | --- | --- | --- | --- | --- |
| a) Patients | Yes | No | a) Nature and purposes of study | Yes | No |
| b) Healthy subjects | Yes | No | b) Procedures to be followed including alternatives used | Yes | No |
| c) Minors or persons under guardianship | Yes | No | c) Physical risks | Yes | No |
| **2. Does the study involve:** |  |  | d) Private questions | Yes | No |
|  |  |  | e) Invasion of the body | NA | |
| a) Physical risks to the subject | Yes | No | f) Benefits to be derived | Yes | No |
| b) Social Risks | Yes | No | g) Right to refuse, to participate or to withdraw from study | Yes | No |
| c) Psychological risks to subjects | Yes | No | h) Confidential handing of data | Yes | No |
| d) Discomfort to subjects | Yes | No | i) Compensation where there are risks or loss of working time or privacy is involved in any particular procedure | NA | |
| e) Invasion of the Body | Yes | No |  |  |  |
| f) Invasion of privacy | Yes | No |  |  |  |
| g) Disclosure of information damaging to subjects or others | Yes | No |  |  |  |
| **3. Does the study involve:** |  |  | **5. Will signed consent form/verbal Consent be required:** |  |  |
| a) Use of records, (hospital, medical, death, birth or other). | Yes | No | a) From Subject | Yes | No |
| b) Use of fetal tissue or aborts | Yes | No | b) From parent or guardian (if Subjects are minors) | NA | |
| c) Use of organs or body fluids | Yes | No | **6. Will precautions be taken to protect anonymity of subjects.** | Yes | No |

**Patient Information Sheet**

(For the patients/respondents)

*(Please read the handout in front of patient/respondent and explain in local language and understandable way).*

The objective of this handout is to give you necessary information that will help you to take decision whether you will participate in this research work or not.

1. About the study:

To assess the impact of COVID-19 Infection in Association with ABO Blood Groups. This research work will be conducted by the Department of Medicine, Dhaka Medical College, Dhaka. We want to include you as a study participant after receiving a written consent from you. I will explain you in a moment what are the components and your role in the study.

2. Purpose of the study:

Find out the impact of COVID-19 Infection in Association with ABO Blood Groups.

3. Confidentiality:

The information that we will collect from this research project will be kept confidential unless permitted by you. Information that will be collected from this study will only be used for research purpose. Your personal information will not be disclosed to anyone other than the investigators.

4. Right to refuse or withdraw:

You have all the right to refuse to participate in this study if you do not wish to do so. Refusing to participate will not affect your treatment in any way. You may stop participating in this study at any time you wish.

5. Incentives:

You will not be provided any incentives to take part in this research. You will be given honorium and conveyance expenditure if you are to come for this research work.

6. Risks and discomforts:

There is a slight risk that you may share some personal and confidential information by chance or that you may feel uncomfortable about some of the topics. However, we do not wish this to happen. You may refuse to give answer to any question or any portion of it if you need to do so.

7. Benefits:

You might not get direct benefit from this study. You will get appropriate treatment in this hospital after the diagnosis of your disease. Your participation is likely to help us to acquire more knowledge about this disease which may be of benefit to other patients of our country.

8. Procedure of research:

If you agree, we will enroll you as a study participant and will adopt the following procedures for your participation-

i. We will take signature/thumb impression in the attached consent form in

duplicate and a copy will be returned to you.

ii. We will ask you some questions to fill in a printed Case Record Form.

iii. You will be examined physically for the sake of this study.

If you agree to participate in this study, please sign the attached consent form.

**INFORMED CONSENT FORM**

I, Mr/Mrs/Miss ……………………………………………., hereby giving informed consent willingly to participate in the study to be done by Dr. Reaz Mahmud. I agree to participate in the study voluntarily without any prejudice. I am fully convinced that during study I will not suffer from any serious physical or psychological problems. I am also informed that this study was carried out in the developed countries safely and my participation will bring fruitful result that will be beneficial for most patients in our country. I have right to withdraw myself from this study at any time. I shall not receive any financial benefit. I have understood that my personal information, medical records & laboratory tests will be kept strictly confidential & will be used for research purpose only.

Signature/Thumb impression of participant/Guardian:………………………….

Date: ………………………………

Name: …………………………………………………

Address: ………………………………………………

………………………………………………………….

………………………………………………………….

Signature of witness Signature of Researcher

Date: Date:

Name of witness:

**Part-G**

**Abstract For National Research Ethics Committee (NREC)**

**Project Title:**

Project Title: **Association of ABO blood groups with presentation and outcomes of confirmed SARS CoV-2 infection: A prospective study in the largest COVID-19 dedicated hospital in Bangladesh**

# Purpose of the study:

To observe the susceptibility of COVID-19 infection in association with ABO blood groups..

**Methodology**:

1. **METHODOLOGY:**

Study type: Prospective cohort

Sample size:

Proportions to cure from COVID-19 by day-12 in group-1(blood group A) and group-2 (blood group Assuming expected B, O, AB) are 0.70 and 0.90 respectively. Thus we need a total of 378 samples at 1:2 ratio, and, that would provide a power of at least 90% at two tailed test where p value less than 0.05 to detect significant difference between the groups. Considering 10% dropout, we need 416 samples in total.

$$n=\frac{r+1}{r}\frac{p^{*}\left( 1-p^{*} \right){(Z_{\beta}+Z_{\frac{\alpha}{2}})}^{2}}{{{(p}_{1}- p_{2})}^{2}}$$

r = ratio of group-1 and group - 2

p*= Average of proportion

Z_α/2_ = Level of significance

Z_β_ = Power of the test

p_1_ - p_2_= Effect size

p_1_ = Proportion in group - 1

p_2_ = Proportion in group - 2

**Sampling: Consecutive sampling.**

Estimated study date: June, 2020

Estimated primary completion date, August 2020

Estimated study completion date: September, 2020

**Inclusion Criteria:**

- At least 18 years of age
- COVID-19 infection, confirmed by polymerase chain reaction (PCR) test
- Mild to moderate COVID-19 infection
- Able to provide informed consent

**Exclusion Criteria:**

- Not willing to participate.
- Discontinue duties for any reason during the specified period
- Presence of haemoglobinopathies or other blood disorders.

**Research instruments:**

1. Informed consent form.

2. Case record form

**Primary Outcome Measure:**

- 1. Duration required to have clinical improvement

[Time Frame: 1 months]

**Secondary Outcome Measure:**

1. Conversion to the next level of severity

Time Frame: 1 month]

1. Persistent positivity 14 days after initial positivity

[Time Frame: 14 days]

1. Severity of the disease

[Time Frame: 1 month]

1. Development of post COVID syndrome

[Time Frame: 1 month]

**Data collection technique:**

Data will be collected by assigned trained data collectors (Physician).

Patient will be enrolled according to defined inclusion and exclusion criteria in the current research. Informed written consent will be obtained from the participants. Each participants participating in the trial will be uniquely identified, and information such as his name, address is recorded in the trial 'subject number list'.

**Data analysis**:

To compare the groups one way ANOVA was done for continuous variable, Chi square for categorical variables. For outcome assessment relative risk (RR) with 95% CI was measured for qualitative variable categorizing blood group-A and non-A (Group-B, AB, O). Survival analysis by Kaplan-Meier curve was done for the duration of recovery.

**Observation and Results:**

Result of the study and statistical analysis will be presented by tables, figures, graphs, diagrams, charts and photographs. All these would have own legends (i.e. title) and will be serially numbered.

**Discussion:**

Discussion will be done on the basis of result obtained from the study and comparing with similar studies done at home and abroad.

**Summary:**

Summarization will be drawn after discussion.

**Conclusion:**

Conclusion would be drawn depending upon the results and discussion

**Conflict of interest:** None

**Conflict of interest:** None

Project Title: **Association of ABO blood groups with presentation and outcomes of confirmed SARS CoV-2 infection: A prospective study in the largest COVID-19 dedicated hospital in Bangladesh**

**Case Record Form**

**Patient ID**: ……………

**Demography**

1. Name: 2. Age: 3. Sex: M / F

4. Address:

5. **Epidemiological link**: Y / N

5. Mobile No:

6. Residency: Urban / Rural

7. Smoker: Y / N

8. Marital Status: Married / Single

9. Education: Literate / Illiterate

Date of starting symptoms

Date of becoming Covid positive

Hospital Admission Date

**Clinical Feature**

| Trait |  | Follow-up  Yes-1, No-2 | | | | | | | | | | | | |
| --- | --- | --- | --- | --- | --- | --- | --- | --- | --- | --- | --- | --- | --- | --- |
|  | On Admission | D2 | D3 | D4 | D-5 | D-6 | D-7 | D-8 | D-9 | D-10 | D-11 | D-12 | D-13 | D-14 |
| Fever | Yes No |  |  |  |  |  |  |  |  |  |  |  |  |  |
| Cough | Yes No |  |  |  |  |  |  |  |  |  |  |  |  |  |
| Running nose | Yes No |  |  |  |  |  |  |  |  |  |  |  |  |  |
| Sputum | Yes No |  |  |  |  |  |  |  |  |  |  |  |  |  |
| Respiratory distress | Yes No |  |  |  |  |  |  |  |  |  |  |  |  |  |
| Sore throat | Yes No |  |  |  |  |  |  |  |  |  |  |  |  |  |
| Hoarseness of voice | Yes No |  |  |  |  |  |  |  |  |  |  |  |  |  |
| Chest pain | Yes No |  |  |  |  |  |  |  |  |  |  |  |  |  |
| Diarrhoea | Yes No |  |  |  |  |  |  |  |  |  |  |  |  |  |
| Vomiting | Yes No |  |  |  |  |  |  |  |  |  |  |  |  |  |
| Anosmia | Yes No |  |  |  |  |  |  |  |  |  |  |  |  |  |
| Anorexia | Yes No |  |  |  |  |  |  |  |  |  |  |  |  |  |
| Headache | Yes No |  |  |  |  |  |  |  |  |  |  |  |  |  |
| Confusion | Yes No |  |  |  |  |  |  |  |  |  |  |  |  |  |

**Subsequent follow up**

| Trait | Follow-up  Yes-1, No-2 | | | | | | | | | | | | | | | |
| --- | --- | --- | --- | --- | --- | --- | --- | --- | --- | --- | --- | --- | --- | --- | --- | --- |
|  | Day-15 | D16 | D17 | D18 | D-19 | D-20 | D-21 | D-21 | D-23 | D-24 | D-25 | D-26 | D-27 | D-28 | D-29 | D-30 |
| Fever |  |  |  |  |  |  |  |  |  |  |  |  |  |  |  |  |
| Cough |  |  |  |  |  |  |  |  |  |  |  |  |  |  |  |  |
| Running nose |  |  |  |  |  |  |  |  |  |  |  |  |  |  |  |  |
| Sputum |  |  |  |  |  |  |  |  |  |  |  |  |  |  |  |  |
| Respiratory distress |  |  |  |  |  |  |  |  |  |  |  |  |  |  |  |  |
| Sore throat |  |  |  |  |  |  |  |  |  |  |  |  |  |  |  |  |
| Hoarseness of voice |  |  |  |  |  |  |  |  |  |  |  |  |  |  |  |  |
| Chest pain |  |  |  |  |  |  |  |  |  |  |  |  |  |  |  |  |
| Diarrhoea |  |  |  |  |  |  |  |  |  |  |  |  |  |  |  |  |
| Vomiting |  |  |  |  |  |  |  |  |  |  |  |  |  |  |  |  |
| Anosmia |  |  |  |  |  |  |  |  |  |  |  |  |  |  |  |  |
| Anorexia |  |  |  |  |  |  |  |  |  |  |  |  |  |  |  |  |
| Headache |  |  |  |  |  |  |  |  |  |  |  |  |  |  |  |  |
| Confusion |  |  |  |  |  |  |  |  |  |  |  |  |  |  |  |  |

**Obsrvation**

| Trait |  | Follow-up  Yes-1, No-2 | | | | | | | | | | | | |
| --- | --- | --- | --- | --- | --- | --- | --- | --- | --- | --- | --- | --- | --- | --- |
|  | On Admission | D2 | D3 | D4 | D-5 | D-6 | D-7 | D-8 | D-9 | D-10 | D-11 | D-12 | D-13 | D-14 |
| Temperature |  |  |  |  |  |  |  |  |  |  |  |  |  |  |
| Pulse rate |  |  |  |  |  |  |  |  |  |  |  |  |  |  |
| BP |  |  |  |  |  |  |  |  |  |  |  |  |  |  |
| Respiratory Rate |  |  |  |  |  |  |  |  |  |  |  |  |  |  |
| Oxygen saturation |  |  |  |  |  |  |  |  |  |  |  |  |  |  |
| Co- Morbidity | DM/HTN/ IHD/ HF/ CKD/CLD/Asthma/COPD/Malignancy/ CTD | | | | | | | | | | | | | |

**Subsequent observation:**

| Trait | Follow-up  Yes-1, No-2 | | | | | | | | | | | | | | | |
| --- | --- | --- | --- | --- | --- | --- | --- | --- | --- | --- | --- | --- | --- | --- | --- | --- |
|  | Day 15 | D16 | D17 | D18 | D-19 | D-20 | D-21 | D-22 | D-23 | D-24 | D-25 | D-26 | D-27 | D-28 | D-29 | D-30 |
| Temperature |  |  |  |  |  |  |  |  |  |  |  |  |  |  |  |  |
| Pulse rate |  |  |  |  |  |  |  |  |  |  |  |  |  |  |  |  |
| BP |  |  |  |  |  |  |  |  |  |  |  |  |  |  |  |  |
| Respiratory Rate |  |  |  |  |  |  |  |  |  |  |  |  |  |  |  |  |
| Oxygen saturation |  |  |  |  |  |  |  |  |  |  |  |  |  |  |  |  |

**Investigations Profile**

| **Trait** | **Value** |  |  |  |  |  |
| --- | --- | --- | --- | --- | --- | --- |
|  | **Admission** | **D-3** | **D-5** | **D-7** | **D-10** | **D-14** |
| **Hb** |  |  |  |  |  |  |
| **WBC** |  |  |  |  |  |  |
| **Neutrophil** |  |  |  |  |  |  |
| **Lymphocyte** |  |  |  |  |  |  |
| **Platelet** |  |  |  |  |  |  |
| **ESR** |  |  |  |  |  |  |
| **CRP** |  |  |  |  |  |  |
| **RBS** |  |  |  |  |  |  |
| **Creatinine** |  |  |  |  |  |  |
| **SGPT** |  |  |  |  |  |  |
| **D-Dimer** |  |  |  |  |  |  |
| **Na** |  |  |  |  |  |  |
| **K** |  |  |  |  |  |  |
| **ECG** |  |  |  |  |  |  |
| **Covid-19**  **RT-PCR** |  |  |  |  |  |  |

**Radio-Imaging**

| **Trait** | **Findings** |
| --- | --- |
| Chest X-Ray  CT Chest | Normal/ Consolidation ( unilateral or bilateral) / Patchy opacity  Consolidation/ Ground Glass Opacity/nodule/ multifocal |

Post COVID symptoms:

Principal Investigators: ……………….. Date:
